# Poster Session II - A296 CULTURALLY RELEVANT DIETARY RECOMMENDATIONS IN IBD - AN UNMET NEED

**DOI:** 10.1093/jcag/gwaf042.296

**Published:** 2026-02-13

**Authors:** J Buttar, S Pan, N Haskey, V Kitchen, N Fu

**Affiliations:** University of British Columbia, White Rock, BC, Canada; University of British Columbia, White Rock, BC, Canada; Biology, The University of British Columbia, Vancouver, BC, Canada; University of British Columbia, White Rock, BC, Canada; The University of British Columbia, Vancouver, BC, Canada

## Abstract

**Background:**

Adherence to guideline-recommended dietary regimens are an integral component of management for patients with Inflammatory Bowel Disease. Current guidelines recommend specific dietary regimens for patients to help achieve and maintain remission. Despite this, there remains a gap in adherence to these recommendations, which may be driven by the cultural diversity and diet preferences of the IBD patient cohort.

**Aims:**

This literature review aims to compile current dietary recommendations for patients with IBD and evaluate their adaptability to diverse ethnic groups. It hopes to illustrate the paucity of data available that is culturally relevant and practical for ethnic populations to follow.

**Methods:**

A scoping literature review was performed on EMBASE and MEDLINE using the COVIDENCE platform. Both databases were searched from inception to Aug 31, 2025. Two independent reviewers screened all eligible manuscripts first with a title review followed by a full text review. Extraction of data was performed by both reviewers and involved qualitative metrics including patient cohort, disease activity, dietary recommendation with intended clinical response and mention of cultural adaptability to ethnic groups.

Inclusion criteria:

-A guideline, consensus statement, review article or formal position document.

-A type of inflammatory bowel disease (Crohn’s disease, ulcerative colitis)

-A tangible recommendation for dietary intervention

Exclusion criteria:

-Parenteral or nasogastric / nasoduodenal forms of nutrition

-Any non-solid medium of nutrition (powders, supplements, vitamins)

-Any original research, abstracts or expert opinion articles not included in a guideline or consensus statement.

-Pediatric populations

**Results:**

348 manuscripts were identified for title review, of which 62 underwent full text review. Of these, 28 studies were included for data extraction. Only one article included culturally relevant material for ethnic populations. Current recommendations include a Mediterranean diet for maintenance of remission, a low FODMAP diet for symptom relief, and a Crohn’s disease Exclusion diet (with partial enteral nutrition) as a means for remission induction. Other dietary regimens, including anti-inflammatory diet, IGG4 exclusion diet, specific carbohydrate diet were mentioned with low levels of supportive evidence.

**Conclusions:**

Multiple consensus statements from national and international gastroenterology organizations emphasize the importance of diet in remission maintenance and as a contributor to induction, however there remains a stark gap in the literature that acknowledges and addresses the ethnic diversity among the IBD patient cohort. Only one study offers a culturally applicable lens to these recommendations. Adherence to dietary recommendations can improve by adapting them to fit the preferences of the various ethnic groups within the IBD cohort.

**Funding Agencies:**

None

